# Muscular Atrophy and Sarcopenia in the Elderly: Is There a Role for Creatine Supplementation?

**DOI:** 10.3390/biom9110642

**Published:** 2019-10-23

**Authors:** Eimear Dolan, Guilherme G. Artioli, Rosa Maria R. Pereira, Bruno Gualano

**Affiliations:** 1 Applied Physiology & Nutrition Research Group, School of Physical Education and Sport, Rheumatology Division, Faculdade de Medicina FMUSP, Universidade de Sao Paulo, 01246-000 Sao Paulo, SP, Brazil; eimeardolan@usp.br (E.D.); artioli@usp.br (G.G.A.); 2 Bone Metabolism Laboratory, Disciplina de Reumatologia, Hospital das Clínicas HCFMUSP, Faculdade de Medicina FMUSP, Universidade de São Paulo, 01246-903 Sao Paulo, SP, Brazil; rosamariarp@yahoo.com

**Keywords:** dietary supplements, ergogenic aids, healthy ageing, muscle, metabolism, bioenergetics, older adults, sarcopenia

## Abstract

Sarcopenia is characterized by a loss of muscle mass, quality, and function, and negatively impacts health, functionality, and quality of life for numerous populations, particularly older adults. Creatine is an endogenously produced metabolite, which has the theoretical potential to counteract many of the morphological and metabolic parameters underpinning sarcopenia. This can occur through a range of direct and indirect mechanisms, including temporal and spatial functions that accelerate ATP regeneration during times of high energy demand, direct anabolic and anti-catabolic functions, and enhanced muscle regenerating capacity through positively impacting muscle stem cell availability. Studies conducted in older adults show little benefit of creatine supplementation alone on muscle function or mass. In contrast, creatine supplementation as an adjunct to exercise training seems to augment the muscle adaptive response to the training stimulus, potentially through increasing capacity for higher intensity exercise, and/or by enhancing post-exercise recovery and adaptation. As such, creatine may be an effective dietary strategy to combat age-related muscle atrophy and sarcopenia when used to complement the benefits of exercise training.

## 1. Introduction

Creatine is an endogenously produced metabolite that has gained large popularity as a dietary supplement due to its fundamental role in human health and physical performance. Its metabolic contributions are many and wide-ranging, and it is one of the most widely used and effective sport supplements available today [1]. Although research interests have traditionally and primarily focused on the potential of creatine to enhance muscle function during high-intensity exercise, evidence of its efficacy in other tissues including the bone [2] and the brain [3] is emerging. In addition, while its role as an ergogenic agent in specific sporting modalities is well-supported by scientific evidence [4], recognition of its potential as a therapeutic agent in a wide range of conditions is increasing [5]. One such condition is age-related sarcopenia. Herein, we will describe the mechanisms through which creatine may theoretically combat age-related reductions in muscle mass, quality, and function, thus ameliorating the negative health consequences of sarcopenia. Additionally, we will review empirical evidence on the efficacy of this supplementation strategy in older adults prior to making practical recommendations to facilitate clinicians and practitioners to make evidence-based decisions related to the potential application of this dietary strategy.

## 2. Sarcopenia and Muscle Atrophy

Sarcopenia is defined as “a progressive and generalised skeletal muscle condition that is associated with increased likelihood of adverse outcomes including falls, fractures, physical disability and mortality” [6,7]. This complex and multi-factorial condition can occur acutely (e.g., following a period of enforced inactivity such as hospitalization), as a comorbidity of other conditions such as cancer, or chronically (e.g., age-related loss of muscle function and mass). It is estimated to impact as much as 10% of the general population aged >60 years [8]. Prevalence rates in non-healthy or dependent older adults are markedly higher, although estimates should be interpreted with caution due to difficulty in assessing such a complex and multi-factorial condition using clinically available screening tools [9]. An important factor in the diagnosis of sarcopenia is whether muscle mass or quality is considered to be the main diagnostic criteria. For many years, sarcopenia was characterized as a loss of muscle mass, and as such, diagnostic criteria was focused on the quantity of lean muscle (as defined by outcomes such as DXA or BIA). However, muscle quantity is a relatively crude indicator of functionality, and over the past decade sarcopenia definitions and diagnostic criteria have evolved to include consideration of muscle quality and function, in addition to, or in place of, quantity [6]. Definition and assessment of muscle quality and function is challenging, however, given the myriad morphological, biochemical, and functional aspects that constitute this parameter [10].

As understanding of the complexity and consequences of sarcopenia evolves, so too does the effectiveness of diagnosis and treatment options. Health-care priorities are shifting from disease to function-centered models. As such, interest in lifestyle, rather than pharmacological interventions (e.g., those that focus on exercise or nutritional habits) are ever-increasing in popularity, and evidence in support of this approach is very promising [11,12]. In relation to the treatment and management of age-related sarcopenia, it is widely accepted that exercise training (and resistance programs in particular) should comprise a fundamental component of treatment and management strategies [13]. However, the role of nutritional factors, and more specifically the contribution of isolated nutrients, is contentious [12]. Creatine is one such nutrient that may have an important therapeutic role to play in sarcopenia management.

## 3. Mechanisms of Creatine Action, and its Potential Role in the Pathophysiology of Sarcopenia

The underlying pathophysiology underpinning the loss of muscle mass and quality that characterizes sarcopenia is multi-factorial and complex. An in-depth description of these factors is beyond the scope of this piece, but a number of comprehensive reviews are available on the topic [10,14]. Briefly, a myriad morphological and metabolic adaptations occurring at the molecular, cellular, and systemic levels combine to reduce muscle anabolic and regenerative activity, while increasing catabolic processes, resulting in a net loss of muscle mass, quality, and function [15]. Theoretically, creatine (methyl-guanidine-acetic acid) has the potential to counteract many of the morphological and metabolic parameters underpinning sarcopenia, and so supplementation in affected individuals has generated considerable research and clinical interest. This can occur through a range of direct and indirect mechanisms, including roles in bioenergetics, direct anabolic and anti-catabolic functions, and enhanced muscle regenerating capacity through positively impacting muscle stem cell availability [2]. Consensus has yet to be reached, however, as to which (if any) of these mechanistic functions are most likely to meaningfully impact those impacted by sarcopenia.

Sarcopenia often results in loss of muscle function; therefore, strategies to enhance the ability of the muscle to generate strength/power are of clinical relevance in aging. An important role of creatine in excitable tissues is to allow for accelerated ATP regeneration during times of increased energy demand, thus allowing for the maintenance of muscle power output and contraction. This can be achieved through both temporal and spatial functions. The phosphorylated form of intracellular creatine (phosphorylcreatine; PCr) acts as a readily available source of cellular phosphate, which on dephosphorylation liberates a phosphate molecule that can then be used to rephosphorylate ATP from ADP. This reversible two-step reaction provides a rapid means of ATP regeneration, and so is particularly useful during times of accelerated ATP demand (e.g., rapid muscle contraction). Additionally, creatine has a role to play in the spatial transfer of energy between sites of high production (e.g., the mitochondria) and sites of high demand (e.g., the sarcomeres) [16]. Oxidatively synthesized mitochondrial PCr can be “shuttled” to cellular locations with a high ADP: ATP ratio and used to rephosphorylate ATP, allowing continuation of cellular work. Termed the “phosphorylcreatine energy shuttle system”, this spatial transfer of energy is facilitated by the presence of distinct creatine kinase isoforms in specific subcellular locations throughout the cell [17]. The spatial shuttling of PCr is more efficient than simply shuttling ATP between cellular sites, because PCr and Cr have faster diffusion rates than do ATP and ADP, and so can move more quickly around the cell. Additionally, the mitochondrial phosphorylation of PCr ensures a higher mitochondrial ADP concentration, which in itself acts as a potent stimulus for continued oxidative phosphorylation [16]. Simultaneously, the availability of PCr at sites of high energy demand reduces ADP concentration at these sites, reducing ADP-mediated CA^2+^ leak from the sarcoplasmic reticulum, thus maintaining force producing capacity [18]. Creatine supplementation has also been reported to increase intramuscular glycogen storage [19].

Creatine also has the potential to counteract sarcopenia and muscle atrophy through a range of direct anabolic and anti-catabolic pathways [20], which may be stimulated, at least in part, by the cellular swelling that occurs due to creatine’s osmotic action. Creatine uptake occurs via a specialized 2Na^+^:1Cl^−1^ creatine cotransporter (CrT solute carrier SLC6A8) [21], and the resultant increase in intracellular fluid volume may contribute towards activation of these anabolic pathways [22]. A large-scale investigation, using both global and targeted assessments of skeletal muscle gene expression and protein content, reported that short-term (10 days) of creatine supplementation stimulated a wide range of genes involved in protein and glycogen synthesis, cytoskeleton remodeling, and signal transduction [23]. These influences occurred independently of exercise, and so represent direct actions of creatine. Similarly, other anabolic growth and signaling factors, including IGF I, II, and phosphorylated 4E-BP1 have been reported to be augmented following a period of creatine supplementation, both in isolation [24] and when supplementation was combined with resistance training [25]. Creatine may also function as an anti-catabolic agent, which is particularly relevant when considering its potential to combat sarcopenia, given that increased catabolism contributes to net muscle loss in those affected [14]. Parise, et al. [26] reported that acute creatine monohydrate supplementation did not affect muscle fractional synthetic rate, but reduced leucine oxidation and the rate of plasma leucine appearance, leading the authors to conclude that creatine supplementation had a direct anti-catabolic effect. In support of this, decreased myofibrillar protein degradation, assessed by 3-methylhistidine, was reported in response to creatine supplementation, alongside resistance training in two groups of older men [27,28].

The direct influences of creatine on muscle may also relate to its anti-oxidant capacity. Reactive oxygen and nitrogen species are by-products of oxidative metabolism, and in small quantities have important functions in cellular signaling and regulation [29]. But an imbalance between the production and neutralization of reactive species can lead to a state of oxidative stress, which is implicated in a wide-range of chronic conditions such as senescence, Alzheimer’s disease, and various cardiovascular and metabolic disorders [29,30]. Indeed, oxidative stress has been implicated in the development of sarcopenia [31,32], and, as such, the generation of reactive species must be balanced by a system capable of quenching these by-products of oxidative metabolism in order to prevent the occurrence of oxidative stress and subsequently protect against sarcopenia. Experimental evidence suggests that creatine may contribute to redox balance through a variety of direct and indirect mechanisms, a full summary of which is beyond the scope of this review, but which has been described in detail elsewhere [33]. As such, the potential of creatine to act as an anti-oxidant represents a pathway through which it may protect against the negative effects of sarcopenia and muscle atrophy.

Muscle tissue is constantly stressed by the fluctuating demands of its environment, and maintenance throughout the life-span is largely determined by its capacity to adapt and regenerate. This capacity depends on the quantity and activity of myocyte precursors, otherwise known as muscle stem, or satellite, cells [34]. These are reduced in aging, thus reducing the ability of the muscle to respond and adapt to stimuli, resulting in muscle loss and deconditioning. As with many of the mechanisms underpinning the aging process, it is unclear whether age-related satellite-cell reductions are a natural and inevitable consequence of aging, or whether they are a product of the environment to which the muscle is exposed [35]. In-vitro and in-vivo evidence supports a positive effect of creatine on satellite cell differentiation and activity. Incubation of satellite cells in creatine monohydrate increased differentiation [36], while creatine supplementation in a group of rats exposed to compensatory hypertrophy of the plantaris muscle (induced by removal of the soleus and gastrocnemius) resulted in a greater increase in satellite cell number, than compensatory hypertrophy alone [37]. Human models have also demonstrated the potential of creatine supplementation to positively impact satellite cells, with supplementation of creatine monohydrate in conjunction with resistance training reported to augment satellite cell number to a greater extent than training alone [38]. Thus, the potential of creatine to positively impact the regenerating capacity of the muscle through increasing satellite cell number represents yet another mechanism through which supplementation may protect against age-related loss of muscle mass and function.

Considered collectively, it seems that creatine has the potential to positively impact muscle mass and function due to a range of direct anabolic, anti-catabolic, and regenerative actions, as well as to indirectly impact muscle function through its temporal and spatial capacity to accelerate ATP regeneration. Just because creatine has the mechanistic potential to counteract many of the pathways underpinning sarcopenia, however, it does not necessarily follow that supplementation with this nutrient will exert measurable effects on clinically meaningful outcomes such as muscle mass, quality, or functional capacity. Accordingly, a large body of empirical research has been conducted to gauge the efficacy of this nutritional strategy, which we will explore in the forthcoming sections.

## 4. Does Age Impact Creatine Content?

The first study to investigate the influence of creatine supplementation on muscle creatine content in humans reported that those with lowest baseline levels experience the greatest response to supplementation, and that the muscle appears to reach saturation at a level of approximately 140–160 mmol/kg of dry muscle, after which no further gains can be made [39]. Creatine supplementation is therefore likely to be most effective in those with low starting levels. When considering the suitability of creatine to counteract sarcopenia and muscle atrophy in older adults, it is prudent to consider A) whether age reduces creatine content, and B) whether age-related creatine declines (if they exist) are a natural consequence of aging, or whether lifestyle related factors (such as activity level and nutrient intake) exert an influence. If creatine content is reduced in older adults, then supplementation may be a useful strategy to counteract this. If these reductions are caused (at least in part) by lifestyle associated changes, rather than age, per se, then this would have implications for whether or not creatine should be recommended in conjunction with other lifestyle recommendations or as an isolated approach. But the answers to these two questions are far from conclusive. Reduced [40,41], or similar [42,43], creatine contents have been reported between older and younger individuals, and there is no clear consensus about if, or to what extent, age impacts creatine content.

In addition to identifying the influence of age on creatine content, and on response to supplementation, it is essential to also consider underlying causes of reduced creatine in older populations, and more specifically older adults impacted by sarcopenia. Many theories of aging exist, with most falling within two broad categories, namely “programmed” and “damage” theories. These theories are complex and multi-factorial and are described in detail elsewhere [44]. Briefly, programmed theories are underpinned by the assumption that aging follows a biological timetable, and as such is largely inevitable. In contrast, damage (or error) theories are based on a belief that various environmental stressors cause damage at many levels (molecular, cellular and systemic), thus leading to breakdown and aging. In reality, both processes are likely to contribute to senescence, with primary aging underpinned by programmed and inevitable functional losses, the extent and timing of which may be influenced by secondary or acquired loss of function due to damage caused by the environment (including lifestyle habits). In relation to creatine, it is not entirely clear whether reported reductions occur as a direct consequence of aging (programmed reductions), or whether other, potentially modifiable factors, may also exert an influence (damage-based reductions). For example, age-related declines in physical activity, or in nutritional intake (and protein in particular) may cause muscle deconditioning and contribute toward reported creatine losses. Indeed, a reduction in the amount of high-intensity activity undertaken in later life may contribute toward the preferential reduction of type 2 (T2) muscle fibres that has been reported to occur in older adults [45]. Creatine naturally occurs in greater quantities in T2, as opposed to T1 muscle fibres [46], and so age-related reductions in T2 fibres may also indirectly influence muscle creatine content. Additionally, reduced energy and protein intake in later years may also contribute toward age-related reductions in creatine content. Further research is required to more precisely identify A) whether or not reduced creatine content is implicated in the pathophysiology of sarcopenia, and B) if reduced creatine content is a direct consequence of aging, or occurs indirectly due to changes in modifiable factors such as activity level or calorie or protein intake. Interestingly, the limited available evidence indicates that older adults are as amenable to creatine supplementation as are their younger counterparts [43,47,48,49], although large inter-individual variation does exist [43]. Creatine transporter protein content and mRNA were also shown to be similar between older and younger individuals [50], suggesting that the reduced creatine content in older individuals observed in some studies may be explained by other aging-related factors (e.g., changes in diet, physical activity, fiber typing), rather than intrinsic changes in muscle creatine metabolism.

## 5. The Influence of Isolated Creatine Supplementation on Muscle Mass and Function in Aging Populations

There is a robust body of evidence showing that creatine supplementation can increase overall muscle function (e.g., strength, daily activity-related tests, and delayed fatigue) and muscle mass in older individuals [51,52,53,54,55]. However, it is less clear as to whether these benefits are brought about by a direct effect of creatine, or whether they are mediated by exercise training.

Several studies have examined the effects of creatine supplementation without exercise training on muscle function and mass. Most of these studies have failed to show a positive effect of long-term (>30 days) creatine supplementation on lean mass [56,57,58,59], although this was not the case in all studies, with some reporting increased lean mass [53,55]. Substantial variation does exist in both dosing protocol (1 g·d^−1^ to ~20 g·d^−1^) and the length of supplementation (7 days to 2 years), but it is unlikely that these differences alone explain this discrepancy in outcomes. Gotshalk, et al. [53] reported a significant increase in lean body mass (+2.2 kg) assessed by hydrostatic weighing after only seven days of high-dose creatine supplementation, which is unlikely to be accounted for by muscle hypertrophy. Due to the known effect of creatine on water retention, this result can be explained by increased body water; in fact, a significant increase in body mass (1.86 kg) was also shown in this study [53], thereby suggesting that most of the increase in lean mass is explained by increased body water. In the study by Gualano et al. [55], 24 weeks of creatine supplementation resulted in no significant increase in lean mass assessed via DXA, but the group taking placebo experienced a significant decrease in lean mass over the course of supplementation. This means that creatine alone counteracted a decrease in lean mass that accompanied aging. These positive results were not reproduced in several other studies [58,59], including two recent large trials that included 109 [57] and 170 participants [56] who supplemented creatine for 1 and 2 years, respectively. Therefore, longer term creatine supplementation, if not accompanied by resistance training, seems to have little or no effect on muscle mass. This is in line with mechanistic studies using isotopic techniques ([1-(13)C]leucine or [(2)H(5)]phenylalanine) that have failed to show any anabolic effects of creatine on muscle protein system at rest, or after resistance exercise, suggesting that creatine alone is not capable of stimulating muscle protein synthesis or blunting protein degradation in skeletal muscle [60,61].

In line with the limited effects of creatine on muscle mass, it also appears that creatine supplementation alone does not substantially improve muscle function, although positive effects have been reported in some parameters [49,51,52,53,59,62,63]. More specifically, most [55,56,57,58,62] but not all [51,53] studies showed no effect of creatine supplementation on maximal strength. On the other hand, strong evidence indicates that creatine supplementation can delay muscle fatigue [49,51,59,62,63]. Interestingly, Wiroth et al. [63] reported that creatine improved performance in sedentary but not trained old men, thereby providing evidence that fitness level might have some influence on the beneficial effects of creatine. In relation to functional tests related to activities of daily living (e.g., timed-stands and timed up-and-go), most [51,55,56,57] but not all [52,53] studies failed to show a positive effect of creatine supplementation alone on functional performance.

Altogether, these results indicate that creatine alone is likely to result in little or no benefit for muscle strength, muscle mass, and functional performance, but might have some positive effects on muscle fatigue. As discussed in the following section, exercise appears to be a major mediator of the beneficial effects of creatine supplementation in older adults. 

## 6. The Influence of Creatine Supplementation in Combination with Exercise Training on Muscle Mass and Function in Aging Populations

It is widely accepted that exercise training (and resistance training in particular) is an effective strategy to attenuate age-related declines in muscle mass and quality, and as such, clinical practice guidelines promote the inclusion of physical training in sarcopenia treatment and management programs [64]. The ergogenic influence of creatine supplementation on the ability of the muscle to perform high-intensity activities is well-recognized [4], as is its ability to augment the hypertrophic response to resistance training [65]. These findings underpin creatine’s popularity as an ergogenic aid, and it is one of the most widely used dietary supplements used by athletes competing in a wide variety of sports [66]. However, can creatine also augment the benefits of resistance training in older adults? This question has been explored in a number of investigations, and the results were subsequently statistically combined by meta-analysis. In 2014, DeVries & Phillips [67] meta-analyzed 10 randomized, placebo-controlled trials (comprising 357 participants, from 55–71 years), all of which investigated whether creatine supplementation could augment the influence of resistance training on body composition, strength and functional performance in older adults. The authors reported a greater effect of creatine supplementation during resistance training compared to resistance training alone on total and lean body mass, leg press, chest press, and functional performance assessed by the 30s second chair stand test, but no effect on fat mass, knee extension, bicep curl, peak isometric, or isokinetic torque. They also urged caution in the interpretation of these results, given the small number of studies available, along with substantial variation in participant characteristics and training protocols. Concurrently, Candow, et al. [68] conducted a similar review. They included 13 investigations, comprising 423 participants. In common with Devries & Phillips [67], Candow, et al. [68] also reported a larger increase in lean mass and chest press strength in the creatine, compared to the placebo group, but in contrast they observed no effect on leg press strength. The results of both of these investigations were certainly encouraging, but results were also somewhat equivocal, particularly in relation to the influence of creatine supplementation on lower body strength, an outcome that may be particularly relevant for maintaining locomotion, and therefore independence, in individuals suffering from sarcopenia. The authors of both reviews urged caution in their interpretation due to the small number of studies available, along with substantial heterogeneity in study population and in the characteristics of the training protocol under investigation. Research in this area substantially increased in the coming years, however, and in 2017 Chilibeck, et al. [69] conducted an updated meta-analysis, including almost double the number of original RCTs than was available for either of the previous two. Based on 22 original studies (comprising 721 participants), the authors reported that creatine supplementation significantly increased lean mass gains (1.37 kg; 95%CI: 0.97–1.76) when compared to the placebo condition. Additionally, greater increases in both upper (chest press) and lower (leg press) body strength were identified in the pooled analysis of those supplemented with creatine during the training program.

As described in Section 3, the influence of creatine supplementation alone on indices of muscle strength and function in older adults is not particularly convincing. But evidence of its efficacy to augment the positive effects of resistance training is stronger. It is not clear which, of the many potentially anabolic or anti-catabolic pathways described in Section 2 underpins this response. It does seem, however, that creatine is at its most effective when combined with a training stimulus, and thus its main influence may come from an ability to enhance the capacity of the body to undertake higher intensity activities, allowing a greater volume or intensity of training to be undertaken. In support of this, Syrotuik, et al. [70] investigated if creatine supplementation would provide any additional benefit to a resistance training program, if volume and intensity of training sessions were controlled, thus preventing the participants (healthy, young, resistance-trained males) from individually maximizing their training stimulus. In line with their hypothesis, the authors observed no additional influence of creatine supplementation over resistance training alone, and concluded that creatine’s ergogenic influence may be due to its ability to enhance training. Although the lack of a positive control in the study by Syrotuik, et al. [70] was a limitation, when considered collectively, the available evidence indicates that creatine supplementation is most efficacious when used as an adjunct to resistance training, rather than as an isolated therapeutic strategy, and that this is true for older, as well as younger individuals.

## 7. Perspectives and Concluding Remarks

**Figure 1:**
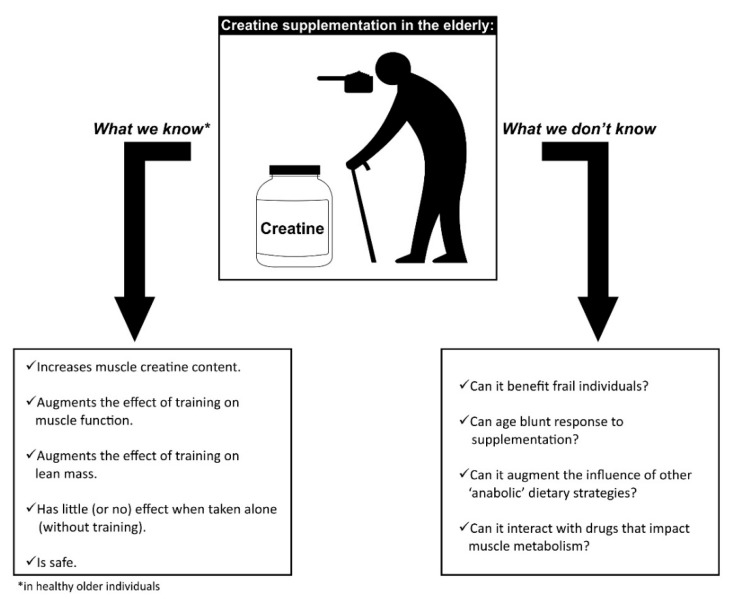
Overview of the state of knowledge about the influence of creatine supplementation on muscle mass and function in older adults.

Even though creatine is one of the most studied dietary supplements, there are still some open questions to be explored, particularly in the elderly (as summarized in Figure 1). It is important to highlight that many of the mechanistic findings described in Section 2 were observed in young men, and replication of these studies in older adults are required to identify if age impacts these responses. This is particularly relevant given that age seems to attenuate responsiveness to exercise interventions [71,72], which may occur due to physiological changes such as reduced sex hormone content and physical activity. Anabolic resistance is a condition characterized by a suboptimal anabolic response exhibited by older individuals [73,74]. Whether this condition may also affect the anabolic properties of creatine supplementation remains to be determined. We have shown that creatine accumulation following creatine supplementation (20 g/day for 7 days) does occur in older individuals, but can be lower than in younger counterparts, especially those under a vegetarian diet [75], which may imply some blunting of responsiveness in older adults.

As previously discussed, the ability of creatine supplementation to improve muscle function is well-described, including in older populations. However, little is known about the role of creatine in increasing creatine content in other tissues besides the skeletal muscle (for a review, see Gualano et al. [2]). Studies involving older individuals need to test whether creatine can also rescue bone loss and cognitive disorders, and to identify the optimal dosing protocol required to elicit these responses. Previous data from our group showed that the classical protocol used to increase creatine in muscle is inefficient to augment creatine in the brain, suggesting that creatine loading is likely tissue-specific [75].

Although most studies show that creatine supplementation does improve muscle function in aging, recent data from our laboratory suggested that creatine supplementation alone for up to two years cannot mitigate muscle dysfunction and lean mass loss in post-menopausal women [56]. This supports the findings from early tracer studies showing that creatine supplementation does not affect muscle protein synthesis or breakdown, suggesting that creatine itself is not anabolic [60,61]. From a clinical standpoint, it appears that creatine should always be prescribed concomitantly with resistance training to promote a beneficial effect in the skeletal muscle. It is also important to investigate whether co-supplementation of creatine with other supplements, such as proteins or amino acids, can lead to greater muscle adaptations in older individuals. The existing studies examining this hypothesis are mainly limited by small sample sizes and short-term follow-ups [76,77], thus limiting interpretation of outcomes. Similarly, the rationale for the co-supplementation of creatine with anti-oxidant supplements, such as coenzyme Q10 or flavonoids (and in combination with physical training) exist [78], although well-controlled studies are required to investigate whether the impact of complementary strategies such as these are additive.

Numerous well-controlled studies involving different populations have shown that creatine is a safe dietary supplement Nonetheless, data in older adults are less abundant, and this may be particularly relevant given that older adults are prone to decreased glomerular filtration rate and may display pre-existing kidney diseases. The monitoring of health aspects in older individuals consuming creatine, particularly in the long run, are clinically and scientifically relevant. A two-year follow-up with postmenopausal women taking 3 g of creatine supplementation did not reveal any adverse effects [56]. Whether this holds true for higher doses remains uncertain. It is also important to consider that many older individuals with various conditions may use medications that potentially affect muscle metabolism, such as glucocorticoids and statins. It is possible that some of these drugs may influence the effectiveness of creatine supplementation. Most of the existing studies involve healthy, older individuals; therefore, caution should be exercised in generalizing the data to older individuals on different drug regimes, or to individuals who are frail.

Collectively, the current literature allows us to conclude that creatine supplementation is a potential dietary intervention to prevent and treat frailty and sarcopenia. However, it is questionable as to whether creatine can benefit older individuals in the absence of resistance training, as creatine seems to act mainly through enhancing the training effects. Novel investigations involving older, frailer populations with appropriate follow-ups and sample sizes are warranted.
